# Economic Analysis of an Image-Based Beef Carcass Yield Estimation System in Korea

**DOI:** 10.3390/ani12010007

**Published:** 2021-12-21

**Authors:** Collins Wakholi, Shona Nabwire, Juntae Kim, Jeong Hwan Bae, Moon Sung Kim, Insuck Baek, Byoung-Kwan Cho

**Affiliations:** 1 Department of Biosystems Machinery Engineering, College of Agricultural and Life Science, Chungnam National University, 99 Daehak-ro, Yuseong-gu, Daejeon 34134, Korea; wcoln@yahoo.com (C.W.); nabwireshona@o.cnu.ac.kr (S.N.); biosch@o.cnu.ac.kr (J.K.); 2 Department of Economics, Chonnam National University, Gwangju 61186, Korea; jhbae@chonnam.ac.kr; 3 Environmental Microbial and Food Safety Laboratory, Agricultural Research Service, United States Department of Agriculture, Powder Mill Road, BARC-East, Bldg 303, Beltsville, MD 20705, USA; moon.kim@usda.gov (M.S.K.); insuck.baek@usda.gov (I.B.); 4 Department of Smart Agriculture Systems, College of Agricultural and Life Science, Chungnam National University, 99 Daehak-ro, Yuseong-gu, Daejeon 34134, Korea

**Keywords:** cost-benefit analysis, automatic grading, slaughterhouse, sensitivity analysis

## Abstract

**Simple Summary:**

Carcass grading is a vital process in the slaughterhouse and is used for the quantification of the overall value of carcasses. Since carcass grading is often performed manually by a team of grading experts, it is subject to human limitations which result in inconsistency and limited operation speed. Considering this, an automatic beef carcass yield estimation system capable of predicting 23 key yield parameters was developed. However, just like any freshly introduced system, analysis of the economic impact of the grading system is vital before deployment in any slaughterhouse business. In this study, a thorough economic analysis to justify deploying the developed beef carcass grading system in a standard slaughterhouse in South Korea was performed through a cost-benefit analysis. The analysis found that the benefits derived from using the developed system outweigh the costs of purchasing and operating the system making the endeavor economically viable.

**Abstract:**

To minimize production costs, reduce mistakes, and improve consistency, modern-day slaughterhouses have turned to automated technologies for operations such as cutting, deboning, etc. One of the most vital operations in the slaughterhouse is carcass grading, usually performed manually by grading staff, which creates a bottleneck in terms of production speed and consistency. To speed up the carcass grading process, we developed an online system that uses image analysis and statistical tools to estimate up to 23 key yield parameters. A thorough economic analysis is required to aid slaughterhouses in making informed decisions about the risks and benefits of investing in the system. We therefore conducted an economic analysis of the system using a cost-benefit analysis (the methods considered were net present value (NPV), internal rate of return (IRR), and benefit/cost ratio (BCR)) and sensitivity analysis. The benefits considered for analysis include labor cost reduction and gross margin improvement arising from optimizing breeding practices with the use of the data obtained from the system. The cost-benefit analysis of the system resulted in an NPV of approximately 310.9 million Korean Won (KRW), a BCR of 1.72, and an IRR of 22.28%, which means the benefits outweigh the costs in the long term.

## 1. Introduction

The livestock industry accounts for approximately 40% of agricultural production in Korea [1]. Livestock production in Korea for the second quarter of 2021 was 3.9 million heads, with over 80% of these cattle (3.5 million) raised for beef production, an increase of 3.7% from the previous year [2,3]. The current domestic beef consumption in Korea is 872,800 tons and is projected to increase by over 4% over the next decade. Globally, the consumption figures for beef are projected to increase over the next few decades because of the projected population growth and wealth increase in the developing world [4].

For a reliable and consistent supply of beef to satisfy the consumer market, there is a need to modernize slaughterhouse operations. For this reason, many slaughterhouses use an overhead rail system, which allows for linear and rotational movement of the carcasses, enabling bulk processing [5]. However, most of the operations are performed by humans, which creates a significant bottleneck to the throughput of the slaughterhouse. To speed up operations, many slaughterhouses have incorporated robotics and automation for operations such as cutting, deboning, grading, etc. This not only speeds up the processes but also improves worker safety and quality assurance and reduces the risk of meat contamination through incautious handling [6,7].

Before the beef is sent to market, beef grading is a key requirement of the production pipeline [8]. Due to the steady increase in production and consumption, it is increasingly important to put in place robust grading systems for the determination of the quality of beef. Carcass grading systems endeavor to classify and value the carcass in terms of quantity and quality, commonly expressed in terms of the lean meat yield (LMY) and/or saleable meat yield (SMY) it contains. Beef carcass grading in Korea is conventionally performed at the loin muscle (rib eye) and classified into five grades depending on the degree of marbling, meat color, fat color, firmness of rib eye area, and maturity, in addition to yield [1].

Initially, beef carcass grading was based on visual assessment, which was found to be subjective and inconsistent. This led to the development of automated video image analysis (VIA) technology in the 1980s. VIA technology involves taking images of the carcass and using software to predict quality-related features [9]. Recent technological developments have resulted in the application of noninvasive methods for carcass grading using X-ray tomography [10,11], magnetic resonance imaging [12,13], ultrasound [14,15], and spectroscopy [16], in addition to video image analysis [17,18,19].

Although these technologies are helpful in the prediction of carcass yield characteristics, most are time-consuming, costly, and have low accuracy in the prediction of key yield parameters. Their low performance could be due to limitations in data/image acquisition systems, outdated and insufficient image and data analysis methods, and a limited sample size. These limitations formed the background for the development of an online image analysis-based system for the prediction of beef yield and quality.

The developed system takes advantage of recent image analysis and statistical tools to estimate up to 23 yield parameters (including carcass lean meat percentage (LMP), major cut weight, hot carcass weight (HCW), etc.) with image data captured from two cameras giving medial and dorsal views of a beef carcass after slaughter.

To justify the financial viability of this system in a slaughterhouse, it is important to determine its true value and the benefits and costs associated with its adoption. This emphasizes the importance of a thorough economic analysis to ascertain the importance of every variable subjected to the analysis. Therefore, good strategies are vital in the determination of the items to be considered as costs or benefits, the time periods in which they occur, and the analysis methods employed. Some of the methods for economic analysis of systems include a cost-benefit analysis (the most widespread), a cost-utility analysis, a risk-benefit analysis, and an economic impact analysis.

The cost-benefit analysis (CBA) of an investment is a decision-making tool based on forecasts of variables over a predefined period. These variables from a CBA are affected by various factors whose effects need to be evaluated to determine the viability of the investment, achieved by carrying out a sensitivity analysis. A sensitivity analysis is the process of recalculating outcomes under alternative assumptions to determine the impact of an input variable; it is considered critical to model validation [20]. A sensitivity analysis focuses on evaluating the effects of changes in key variables on the internal rate of return (IRR) or net present value (NPV), widely accepted measures of an investment’s worth. An investment is considered viable when the NPV is positive [21,22].

In order to determine the viability of a project, all aspects (costs, benefits) must be expressed in terms of monetary value. In the cattle industry, CBA has been applied in the development of a model to investigate the economic performance of Scottish beef enterprises [23], the determination of economic efficiency of beef production systems [24], and to understand the financial cost of applying intervention systems to improve color and hence carcass value at grading [25].

This study was therefore conducted to determine the economic viability of the introduction of this novel system into the beef grading pipeline in a standard slaughterhouse (a typical modern slaughterhouse) in South Korea. This study examines the rigors of determining the expected present and future costs, the benefits of procuring the system, and how they feature over the lifespan of the system to provide an informed basis for investment decision making.

## 2. Materials and Methods

The economic analysis process involves the evaluation of costs and benefits, which help us to rank projects based on economic viability for the better allocation of resources. It aims at analyzing the economic and welfare impact of a project [26]. Economic analysis helps to address questions such as: Should the project be undertaken? What will be the fiscal impact of the project? How do we ensure efficiency and equity of cost recovery?

This economic analysis was performed through a series of steps presented in this section. These include determining the project life, discount rate, enumeration of benefits, and costs of procuring the system for a typical slaughterhouse in South Korea. This is followed by the actual CBA and a sensitivity analysis.

### 2.1. Beef Carcass Yield Estimation System

**Figure 1:**
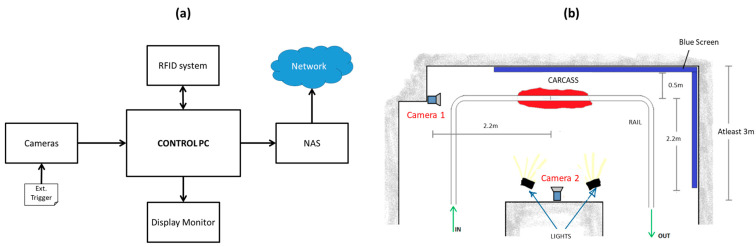
Proposed system: components (**a**) and setup (**b**).

**Figure 2:**
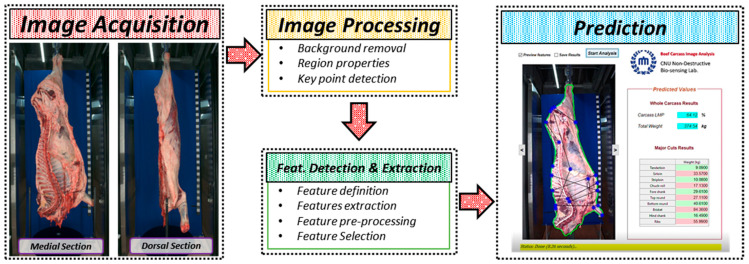
Schematic of operations from image acquisition to final yield predictions.

The beef carcass yield estimation system developed (seen in Figure 1a) consists of two color cameras, a radio-frequency identification (RFID) system, a control PC, and network-attached storage (NAS). When the beef carcass half gets to the detection zone (a predetermined position), it triggers both cameras to capture images of the carcass. The image data are then transferred to the control PC, which runs the image analysis and prediction model and returns the yield results in less than 8 s (see Figure 2). The yield prediction results are then written onto an RFID tag that is unique to each carcass and is attached to the carcass hook. The images and prediction results are also saved (based on the carcass identity read from the RFID tag) on the NAS and can be shared to any PC on the network. For every carcass that is monitored, an overlay of the carcass medial view image and grading results are displayed on the monitor.

The implementation of this system has multiple advantages including a reduction in the number of and/or support for carcass grading technicians, which saves costs and helps ensure the consistency of grading results. The system also provides yield data before actual carcass dissection and can be used to build an online auction database for order management. It results in reduced risk of the contamination that arises from carcass handling since the system is noncontact, and an improvement of yield through breeding optimization using data from the system. Despite the system’s ability to predict yield parameters with a commendable degree of accuracy, it cannot completely replace the grading staff, as some key grading parameters such as marbling still need to be evaluated from the analysis of the rib-eye in Korea (between the 12th and 13th ribs).

### 2.2. Project Life

To determine the lifetime of a project, it is important to determine how long the project is expected to last. The project’s lifetime is determined by how long until an asset/equipment generates more income than it costs to maintain and operate, i.e., how long the net economic value of an asset takes to be reduced to zero. According to the Korean Public Procurement Service (PPS), the persistent period of similar industrial equipment/machinery machines was set to nine years [27], and thus the project life of the system was set to nine years for this study.

### 2.3. Discount Rate

The discount rate is defined as the interest rate used to calculate future cash flows to their present-day value. It is used in the computation of the time value of money, which is instrumental in a CBA, specifically for the calculation of NPV and IRR. According to the Korea Development Institute (KDI) recommendations for evaluating public projects, the discount rate value was set to 4.5% for this project [28].

### 2.4. Costs

The cost is the monetary value of expenditures for supplies, services, labor, products, equipment, etc. that are not available for use anymore. This subsection breaks down the costs that arise from using the system. In a cost-benefit analysis, costs are divided into fixed and variable costs. In the context of this study, fixed costs refer to predetermined expenses that remain the same throughout the project life, regardless of production output, while variable costs refer to expenses that vary directly or proportionally depending on the production output of the project.

#### 2.4.1. Fixed Costs

The fixed costs considered for this project include the initial price of the system, installation costs, and the cost of the space where the system is to be installed. These costs will be incurred once at the start of the project rather than throughout the project life.

##### Initial System Price

The initial price of procuring the system was internally determined by the system development team. This was determined based on the cost of individual system components, research and development, and system software development. The price of procuring the system was set to 79,500,000 Korean won (KRW), which is about 16% of the average cost of the most renowned image-based carcass evaluation technologies (including the VCS2000 (e+v Technology GmbH and Co.KG, Oranienburg, Germany), BCC-3 (Frontmatec Group, Kolding, Denmark), and DEXA (Scott Technology, Dunedin, New Zealand). This makes the system significantly cheaper than the alternatives while having relatively good prediction performance, albeit for fewer yield parameters.

##### System Installation Costs

The cost for installing the system includes all the costs associated with system component setup and calibration, and overhead rail modification to suit the system design. The process of system component setup and calibration was assumed to be accomplished by a team of two technicians for a duration of one week. Based on the average daily wage of technical workers (with expertise in system software development) in South Korea, the cost of system component setup and calibration was determined to be 2,601,210 KRW. The cost for overhead rail modification based on the system design (Figure 1b) was determined to be 8,283,000 KRW (overhead rail cost of 7,030,000 and labor cost of 1,253,000 KRW).

##### Cost of Land/Space

From Figure 1b, the system occupies approximately 17.5 m^2^ (3.5 m × 5 m) in the slaughterhouse. To compute the cost of this space, the locations of 20 slaughterhouses in South Korea were considered in order to determine the unit cost of land (price per m^2^) in these locations [29]. The average price per m^2^ was then multiplied by the system space requirement. The total cost of the land occupied by the system was determined to be 3,441,498 KRW.

#### 2.4.2. Variable Costs

The variable costs considered for this project included system operational costs and system repair and maintenance costs. Most variable costs were incurred annually, except for some repair and maintenance costs, which followed the individual component warranty period.

##### Operational Costs

The operational costs incurred using this system include electrical costs, internet costs, and administrative costs. The electrical costs were computed based on the total system power consumption (approximately 860 W), system operation time, and the annual cost of electricity, which was computed using Equation (1) below. The system operation time was set to 9 a.m.–12 p.m. and 1–6 p.m., which is a total of 8 h per day. The type of electricity consumed by the system was based on the type of electricity consumed by an average scale of the slaughterhouse (consumption: 300 kW or more) in South Korea, which is Industrial B, high-voltage A, option II, as defined by Korea Electric Power Corporation (KEPCO) [30]. Since the electricity tariffs vary depending on the season and the time of day, the total power consumption is a sum of the based power consumed and the additional charges depending on the season and time of day. The total annual cost of electricity was found to be 15,160,339 KRW.

(1) $$\begin{array}{c} C_{\mathrm{total}}=P\left(\mathrm{kW}\right)\times \left[C_{\mathrm{base}}{+C}_{\mathrm{summer}}{+C}_{\mathrm{spring}/\mathrm{fall}}{+C}_{\mathrm{winter}}\right]\\ C_{\mathrm{base}}=8320\;\mathrm{KRW}\;\times \;8\;h\;\times \;5\;\mathrm{days}\;\times \;52\;\mathrm{weeks}\\ C_{\mathrm{summer}}=\left(\left(186.1\;\mathrm{KRW}\;\times \;6\;h\;\right)+(104\;\mathrm{KRW}\;\times \;2\;h)\right)\;\times \;5\;\mathrm{days}\;\times \;13\;\mathrm{weeks}\\ C_{\mathrm{spring}/\mathrm{fall}}=\left(\left(104.3\;\mathrm{KRW}\times 6\;h\right)+\left(73.6\;\mathrm{KRW}\times 2\;h\right)\right)\;\times \;5\;\mathrm{days}\;\times \;22\;\mathrm{weeks}\\ C_{\mathrm{winter}}=\left(\left(161.7\;\mathrm{KRW}\;\times \;3\;h\right)+(104.2\;\mathrm{KRW}\;\times \;5\;h)\right)\;\times \;5\;\mathrm{days}\;\times \;22\;\mathrm{weeks} \end{array}$$

where; C is the cost of electricity and P is the system wattage.

The details of the calculations are that summer lasts from June to August (with two peak and six mid-peak hours), winter from November to February (with five peak and three mid-peak hours), and spring and fall from March to May and September to October, respectively (with two peak and six mid-peak hours).

Since the system stores the images and grades results on NAS, which is accessible by the technical team via the internet, an internet connection with sufficient speed and reliability is required. For this reason, the average internet usage fee from three Korean mobile internet service providers (SKT, KT, and LGU+) was sourced. The average internet rate for each mobile service company differed depending on the speed of the internet. For this study, a 100 Mbps internet connection was selected since it is sufficient, and the annual cost was found to be 270,000 KRW [31].

Administrative costs are the costs incurred by a company that are not directly related to manufacturing but are relevant to the overall profitability of the company. The administrative costs for this project mostly relate to system management (accounting and clerical work) and information technology services. The administrative costs for this project were set to 15% of the fixed costs of the project, amounting to approximately 12,624,009 KRW.

##### Repair and Maintenance Costs

The repair and maintenance costs include the repair and replacement of core system components (PC, cameras, NAS), software support, and replacement of RFID tags.

The core system components such as the PC and the NAS are to be repaired in the fourth and eighth years of the project, a decision based on the manufacturer’s product warranty. The cost of repairing the PC and NAS was set to 25% of their initial cost, resulting in 489,930 and 433,000 KRW, respectively, for each repair period. The cameras had a warranty of four years; therefore, camera replacements were planned in the fourth and eighth years of the project. The cost of replacing the cameras was 6,299,593 KRW for each replacement period. Because of possible exterior and/or interior damage to the RFID tags, 10% of the RFID tags (80 tags) were replaced every year. RFID tag replacement cost 641,200 KRW per year based on a price of 8015 KRW for every tag [32]. A summary of the fixed and variable costs incurred during the project is presented in Table 1 below.

#### 2.4.3. Depreciation Costs

Depreciation refers to the process of allocating an amount less the residual value from the acquisition cost of an asset in a systematic and reasonable manner during the period in which the asset is in operation. It is used to reallocate the cost of a tangible asset over its useful life span. The depreciation costs were calculated using the sum of the years’ digits (SYD) depreciation method [33]. The SYD method is an accelerated method for determining an asset’s expected depreciation over time and assumes the asset loses most of its value at the beginning of its useful life. The SYD method results in a larger depreciation tax shield in the first few years of the asset’s life. Sometimes an asset will lose most of its value toward the beginning of its useful life and will decline more gradually compared to the double-declining balance method. SYD values for each year were determined using Equation (2). For this study, the duration over which depreciation was to occur was set to the project life (nine years), and the system’s salvage value after the nine-year period was set to 5% of the cost of assets. Since the cost of assets (initial system, overhead rail, and land) was 89,937,063 KRW, the salvage value, therefore, was 4,496,853 KRW.

(2) $$\mathrm{SYD}=\frac{\left(\cos t-\mathrm{salvage}\right)\times \left(\mathrm{life}-\mathrm{period}+1\right)\times 2}{\mathrm{life}\times \left(\mathrm{life}+1\right)}$$

where cost is the initial cost of the assets to be depreciated, salvage is the value of the asset at the end of the depreciation, life is the number of periods over which the asset is being depreciated, and period is the period for which the depreciation is to be determined.

### 2.5. Benefits

A benefit is an advantage or profit gained from something. This subsection details the benefits that arise from using the system. For this study, benefits are divided into direct, indirect, and intangible benefits. Indirect benefits are returns from using a system or asset that cannot be directly observed but are nonetheless realized, while direct benefits can easily be observed and quantified.

#### 2.5.1. Direct Benefits

Through the utilization of the system, there is a direct reduction in the burden and fatigue of grading carcass and consequently a reduction in labor. The analysis assumes that, for an average Korean slaughterhouse with two or more grading experts, the introduction of the system will reduce the size of the grading team since yield estimation, a key step in carcass grading, will be performed by the system. It is assumed that the system will be able to directly benefit the slaughterhouse by saving the salary of at least one person from the grading team. This will result in a benefit of 42,509,220 KRW per annum based on the average wages earned by a middle-class quality control worker in South Korea [34].

#### 2.5.2. Indirect Benefits

In South Korea, there are currently over 93,000 beef cattle farmers and over 3.5 million head of cattle. According to statistics, about 43% of the cattle are owned by largescale farmers who own over 100 heads of cattle each [3]. Most farmers have adopted livestock breeding practices to improve the meat yield of their livestock. The impact of these breeding practices has resulted in a general increase of approximately 40 kg in the average weight of cattle over the past 10 years, which is about a 0.45% increase per annum [35]. It also resulted in a reduction in beef cattle growth time/slaughter age (i.e., the percentage of cattle slaughtered at less than 12 months old was 27% in 2014 and increased to 31% in 2021), which reduces the production costs as well [36]. Most evaluation systems for quantifying the impact of breeding practices are based on the body weight and age of animals as the key factors, i.e., the more muscle/meat extracted from the animal, the better [37,38,39]. However, other meat quality factors such as carcass LMP and marbling can be used to evaluate and improve breeding practices.

The system can be used to estimate carcass yield parameters and subsequently share the yield information with the livestock farmers to be used as an evaluation basis for the optimization of the breeding process. This can result in the production of animals with good meat yield, for a shorter growth time, at an optimized breeding cost for the farmer.

Our analysis follows the breeding and slaughter system in South Korea, where farmers grow cattle to full maturity before choosing from three pathways for slaughter. Pathway one (practiced by 4.2% of farmers) involves the farmer paying the slaughterhouse to slaughter, inspect, and grade their cattle for a price of approximately 170,000 KRW, after which they sell the carcass to the highest bidder through auctioning. For pathway two (practiced by 45.9% of farmers), the farmer sells the cattle to a livestock broker who goes through a similar process before selling the meat or carcasses in an auction. Lastly, in pathway three (practiced by 49.9% of farmers), the farmers sell the cattle to a large corporation that also goes through the same process before finding the most suitable market to sell the meat [40,41]. In this setup, most slaughterhouses do not buy cattle directly from farmers but offer slaughter, grading, and sometimes further carcass dissection as services to farmers.

**Figure 3:**
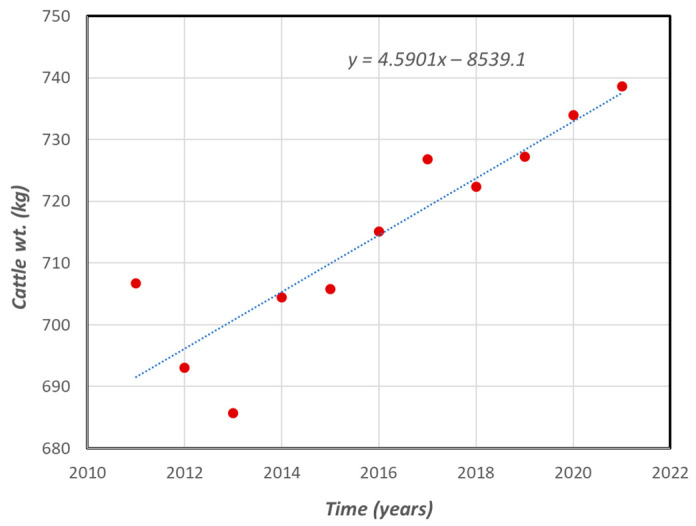
Trend of average weight of live beef cattle produced in Korea since 2011 (source: KOSIS [44]).

To enumerate the benefits resulting from breeding practice optimization due to the shared information between slaughterhouses and cattle farmers, we made the following assumption. According to the Korean Ministry of Agriculture livestock improvement goals [42], the rate at which breeding practices have improved overall cattle live weight has been approximately 0.62% per annum. Based on this value and the actual observed live weight increase over the past 10 years, we decided that using a base rate of cattle improvement of 0.4% per annum is sufficient for our analysis. The average cost of raising a single beef cow in South Korea is about 9.3 million KRW [43], which results in a unit cost of 13,561 KRW per kg since the average weight (W) of a beef cow in Korea is currently approximately 688 kg [44]. Because of breeding improvements, this cost per kg of live cattle weight is bound to reduce over the nine years of the project life, as depicted in Figure 3.

**Figure 4:**
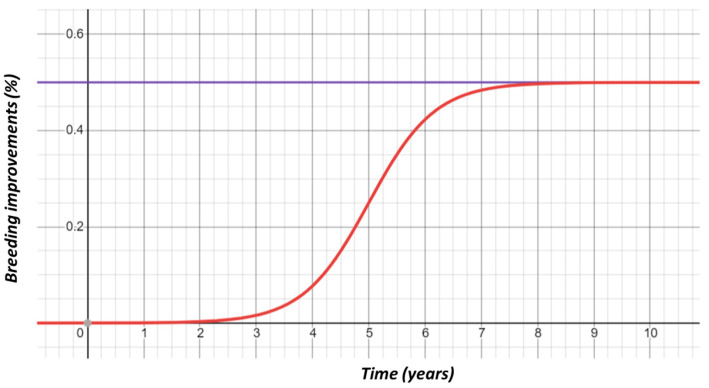
A sigmoid curve used for computing the increase in gross margin over the project’s life.

**Figure 5:**
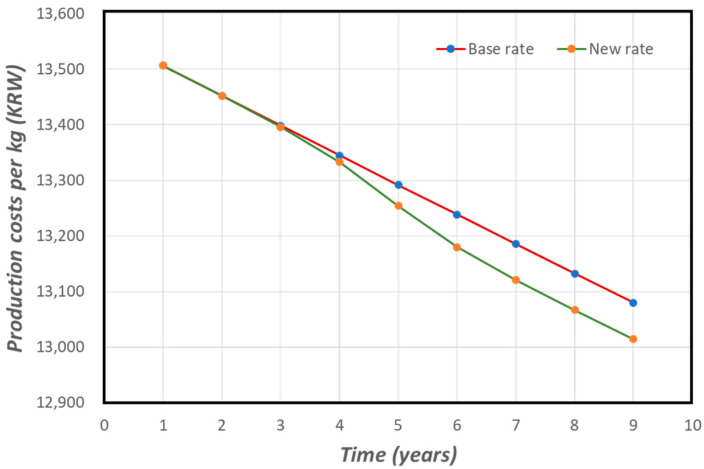
Projected beef cattle production costs using the base rate (based on the national improvement target rate) and a new rate based on the utilization of our system.

The utilization of our system to estimate carcass yield and the subsequent sharing of the yield data with farmers can result in further breeding practice optimization and a reduction in the unit production cost. The resultant unit cost saving was assumed to be a maximum of 0.5% throughout the project life, which would not be significant in the initial years of the project but would increase to a maximum of 0.5% in the final years following a custom sigmoid function (Figure 4). The difference between the new annual unit production cost and the original production cost was the benefit of using the system (Equation (4)). The resultant value was used to calculate the total production costs saved every year given the average number (N) of cattle slaughtered per slaughterhouse was 2798.27 per annum [45], computed using Equation (5) (see Figure 5 for a comparison with the base unit cost).

(3) $$\mathrm{new}\;\mathrm{unit}\;\cos t=\;\mathrm{original}\;\mathrm{unit}\;\cos t\times \frac{0.5\%}{1+e^{\left(-1.7\times \;\mathrm{period}+8.5\right)}}$$

(4) difference=original unit cost−new unit cost 

(5) Savings per year=difference × W × N 

where, period is the specific period for which the gross margin increase is being calculated.

#### 2.5.3. Intangible Benefits

For this study, intangible benefits consist of benefits that directly or indirectly result from the utilization of the system for carcass yield estimation but are difficult to quantify in monetary terms. These include improvements in worker safety and food safety, the creation of an online database, etc.

The introduction of the system to the slaughterhouse would simplify the availability and accessibility of the carcass image data and resultant yield information. The readily available carcass data can be used to create an online database that makes information available to stakeholders (e.g., buyers, governments, and scientists) so they can make informed decisions or build systems upon the data. In Korea, this information can be used by the government to monitor national carcass production in real-time. It can also be used to update the online auction system (currently under implementation in Korea [46]), thus easing decision-making for meat buyers/traders. Since information on carcass yield is available after slaughter and before dissection, this is helpful for management to sort the carcasses for cold storage and to give livestock farmers an accurate price for their cattle based on the predicted yield parameters.

Since the system is noncontact in nature, yield parameters that used to be determined in a hands-on manner can now be assessed automatically. Therefore, the system can reduce the food safety issues that may arise from slaughterhouse handling. Additionally, there is a reduction in workers’ exposure to dangerous tools and equipment (e.g., during weighing and dissection), thus improving worker safety.

### 2.6. Cost-Benefit Analysis Methods

The purpose of a CBA is to determine if the decision to invest in a system is sound and to know if the resultant benefits outweigh the costs, and by how much. All the flows of benefits and costs over time are expressed in terms of their NPV, regardless of whether they are incurred at different times. The CBA techniques considered for the evaluation of the economic feasibility of the system include NPV, BCR, and IRR.

#### 2.6.1. Net Present Value

Net present value (NPV) analysis is commonly used for evaluating the feasibility of a project. Because the costs and benefits arising from a project continue into the future, future benefits and costs should be considered by converting them to present values. In simple terms, NPV refers to the present value of the expected future benefits and costs resulting from the investment [47]. The formula for obtaining NPV is as shown in Equation (6):

(6) $$\mathrm{NPV}=\overset{n}{\underset{t=1}{\sum }}\frac{B_{t}-C_{t}}{{\left(1+r\right)}^{t}}\;\;$$

where t is the termination time under consideration, $C_{t}$ is the cost cash flow under consideration in a specific period, $B_{t}$ is the benefit cash flow under consideration in a specific period and n is the project’s duration period.

#### 2.6.2. Benefit/Cost Ratio, BCR Analysis

BCR refers to the ratio of the present value of the benefits ($PV_{benfits}$) divided by the present value of the costs ($PV_{costs}$) arising from the investment. BCR is a good indicator of the overall value for money of the project. If BCR is greater than one, the project is considered economically feasible for investment [48]. The calculation of BCR is shown in Equation (7):

(7) $$\mathrm{BCR}=PV_{benfits}\;÷\;PV_{costs}\;$$

#### 2.6.3. Internal Rate of Return

The internal rate of return (IRR) is a metric used in financial analysis to estimate the profitability of potential investments. It is defined as the discount rate that makes the NPV of the project zero. Generally, the higher the IRR value, the more desirable an investment is to undertake. If the IRR is greater than the discount rate, the investment is economically valuable. The computation of IRR was performed using Equation (8):

(8) $$\mathrm{NPV}=0=\overset{n}{\underset{t=1}{\sum }}\frac{B_{t}-C_{t}}{{\left(1+\mathrm{IRR}\right)}^{t}}\;\;\;\;$$

where, $C_{t}$ is the net cash inflow during period t and $C_{0}$ is the total initial investment costs.

### 2.7. Sensitivity Analysis

A sensitivity analysis investigates how changes in the assumptions of an economic analysis generally affect the predictions. Ideally, the determined benefits and costs in the cost-benefit analysis include many assumptions and uncertainties. Therefore, a sensitivity analysis is performed to address these uncertainties. The sensitivity analysis identifies changes in the NPV, IRR, and BCR values by changing the critical variables that can affect the economic value within a certain range. For this study, five parameters were considered (the discount rate, initial investment, number of employees let go due to labor automation, the percentage increase in the gross margin due to the optimization of breeding practices, and annual carcass throughput per slaughterhouse) for the economic analysis to be sufficiently sensitive. These parameters were varied while monitoring the changes in NPV, IRR, and BCR values. Further details of the variation of the parameters for the sensitivity analysis are presented in Table 2 below.

## 3. Results

### 3.1. Cost-Benefit Analysis

The results of the economic analysis, including the resultant cash flow and CBA, are summarized in Table 3 below. The CBA is represented by the NPV, BCR, and IRR values resulting from a persistent period of nine years and a discount rate of 4.5%. From Table 4, it can be observed that the project incurred the highest costs (93.8 million KRW) at the beginning of the project’s life. 

From the results in Table 4, it can clearly be observed that the benefits from the project generally increased throughout the project’s life (from 42.7 million KRW in the first year to 168.3 million KRW in the last year). The system’s operational costs generally remained constant at 34.8 million KRW per annum throughout the project life. The total present value of the benefits was calculated to be approximately 1.15 billion KRW, while the total present value of costs was 431 million KRW. From these two values, using Equation (4), the NPV was calculated to be approximately 311 million KRW. The BCR was found to be 1.72, and the IRR value was also found to be 22.28%.

**Figure 6:**
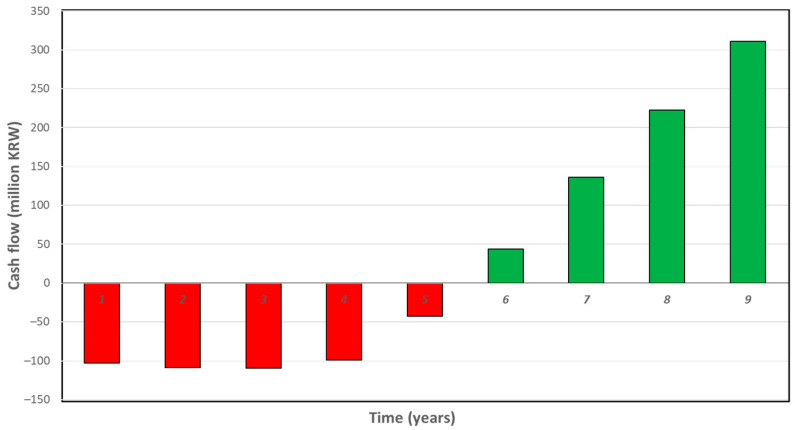
Plot of the project net cash flow over the project’s life.

On further analysis of the present value of the project cash flows over the project’s useful life, it was found that the break-even point (the point at which the project turns profitable) was achieved during the sixth year of the project (which is about half of the project’s life; see Figure 6).

### 3.2. Sensitivity Analysis

**Figure 7:**
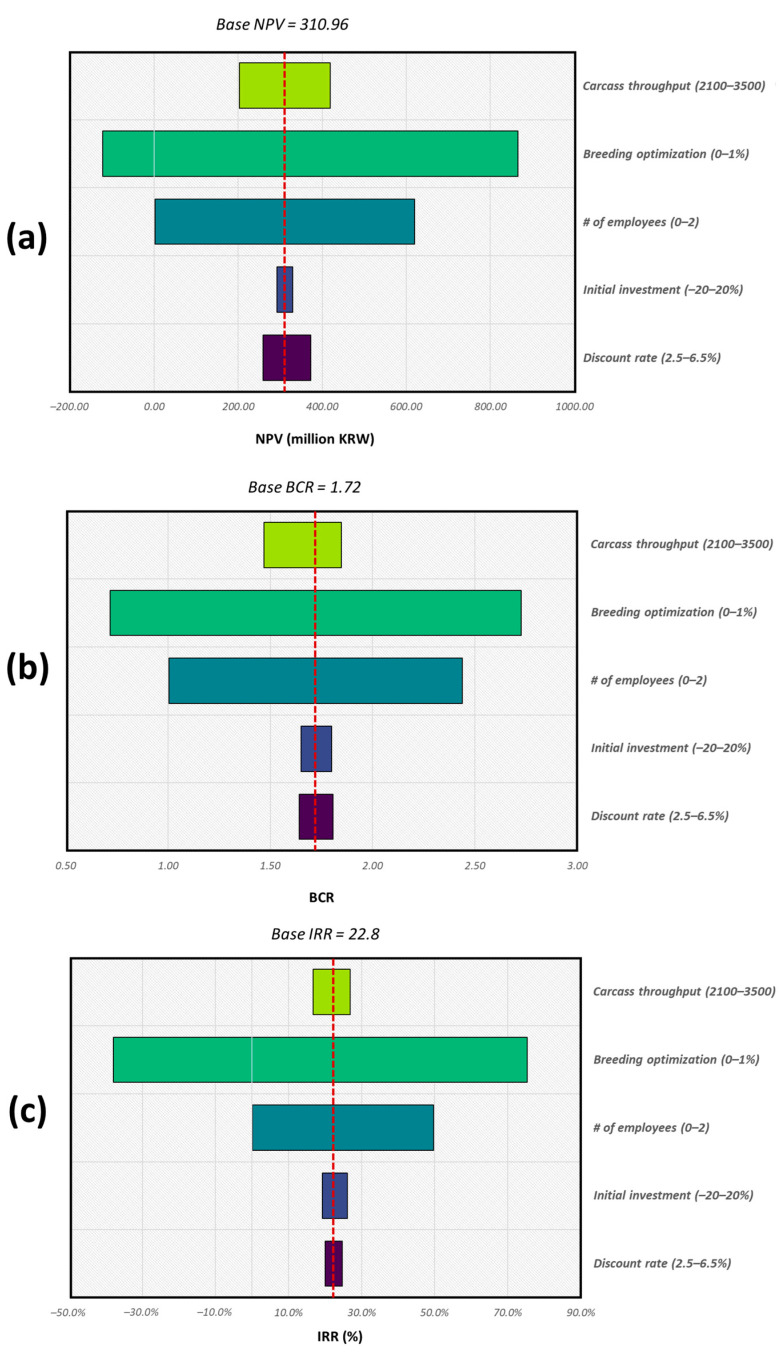
Sensitivity analysis results for each parameter and how they vary: (**a**) NPV, (**b**) BCR, and (**c**) IRR.

The results of the sensitivity analysis are summarized in Figure 7. In this sensitivity analysis, the initial investment, total benefits, number of employees replaced by the system, percentage increase in the gross margin, and average slaughter volume per slaughterhouse per annum were adjusted within specific ranges, as defined in Table 3, while monitoring the NPV, BCR, and IRR values.

With the adjustments, as the discount rate decreases, the NPV, BCR, and IRR increase, and the reverse happens when the discount rate is increased. The NPV, BCR, and IRR values achieved with the discount rate values varying from 2.5% to 6.5% were 258.7 million KRW, 1.64, and 19.98%, and 372.3 million KRW, 1.81, and 24.67%, respectively.

The reduction of the initial investment by 20% resulted in an increase in the NPV, BCR, and IRR values (to 329.7 million KRW, 1.80, and 26.05%, respectively). When the initial investment was increased by 20%, the values of NPV, BCR, and IRR changed to 292.2 million KRW, 1.65, and 19.27%, respectively.

The benefits of the introduction of this system in a Korean slaughterhouse are a reduction in labor costs and subsequent optimization of breeding practices. An increase in both benefits resulted in increases in the NPV, BCR, and IRR values because they directly reduced the costs and increased the benefits of the implementation of the system. When no employees were replaced and costs were saved due to breeding practice optimization being maintained at 0.5%, the project achieved NPV, BCR, and IRR values of 2.0 million KRW, 1.01, and 0.14%, respectively. Conversely, when the benefit of costs saved due to breeding practice optimization was set to 0%, the NPV, BCR, and IRR values were −122.0 million KRW, 0.72, and −38.02%.

Lastly, the effect of increasing the average annual carcass throughput of slaughterhouses resulted in an increase in the values of NPV, BCR, and IRR and vice versa.

## 4. Discussion

### 4.1. Cost-Benefit Analysis

From the results of the project cash flows and CBA as summarized in Table 4, it was observed that the project incurred the highest costs at the beginning of the project’s life, this was because of the purchase of the system and its installation cost. The system operational costs were different in the fourth and eighth year of the project life. This was because of the replacement of system cameras and repairs to the system PC and NAS are incurred. The additional system camera replacement and repair costs total 7.2 million KRW for each of the fourth and eighth year of the project (more details in Table 1).

While the benefits from the project generally increased throughout the project’s life, the rate at which the benefits grew was low in the early years, improved in the middle years of the project, and slowed down when nearing the end of the project. This was mainly because of the choice to reflect the indirect benefits (due to the breeding practice optimization resulting from using the data from our system) to follow a sigmoid function (described in Equation (3)).

From the CBA, the project resulted in a positive value of NPV (approx. 311 million KRW), a BCR of 1.72, and an IRR of 22.28%. Since the achieved value of NPV was positive, BCR > 1, and IRR was greater than the nominal discount rate (4.5%), this means that the benefits far outweigh the costs arising from using the system thus investing in the system would be a good investment with a positive economic return.

Since the project was observed to hit break-even in the 6th year of the project, this further emphasizes the profitability of the project and can be used as a basis to persuade investors.

### 4.2. Sensitivity Analysis

The sensitivity analysis results showed that the decrease in discount rate resulted in increases in the NPV, BCR, and IRR values and vice versa. Lowering the discount rate resulted in an increase in the net present value of the project. This is because most of the benefits of the project increased towards the end of the project life, thus the present value of such benefits was quite high compared to the costs.

The improvement of the NPV, BCR, and IRR values on reduction of the initial costs of investment was because of the reduced costs due to the lower initial investment and reduction on depreciation costs.

The effect of varying the project benefits directly affects the NPV, BCR, and IRR values. On reduction of the project benefits, the NPV, IRR, and BCR values reduce because this directly reduces the net present benefits of the project, the same happens vice versa. Therefore, an attempt to eliminate any of the project benefits resulted in the project not being viable economically.

The reason for the increase in the values of NPV, BCR, and IRR with the increase of the average annual carcass throughput of slaughterhouses is because the increase in carcass throughput directly contributes to the benefit of costs saved due to breeding practice optimization. To investigate further the combined effect of carcass annual slaughter volume and costs saved due to breeding practice optimization on the values of NPV, BCR, and IRR was investigated. It was observed that increasing the annual slaughter volume (in steps of 350 carcasses) was not as impactful as increasing the costs saved due to breeding practice optimization. This implies that, for the system to achieve maximum economic impact, the annual slaughter volume should be increased above the average Korean slaughterhouse annual volume. Most importantly, emphasis should be put on sharing carcass yield data with livestock farmers to optimize breeding processes, reduce unit production costs, and consequently increase production cost savings for the farmers.

A comparison of the impact of the five selected variables on the overall CBA is given in Figure 7. Variation in the initial investment cost had the least impact, followed by the discount rate, then the annual carcass throughput, and lastly the benefits (direct and indirect). The reasons for this trend are the low initial investment costs and variable costs, which are too small to make a significant impact on the overall viability of the project. This is why varying the benefits (gross margin increase and reduction of the number of employees) has such an impact on the values of NPV, BCR, and IRR.

## 5. Conclusions

This study was conducted to determine the economic value and utility of an online image analysis-based beef carcass yield estimation system using a cost-benefit analysis. During the persistent period of this system, the net present value was found to be 310.9 million KRW, the BCR was 1.72, and the IRR was 22.28%. In each of the economic analyses undertaken, the benefits outweighed the costs in the long term. In particular, the investment was predicted to pass the break-even point within four years due to the benefits. As the sensitivity analysis suggests, to maximize the economic effects of the system, the number of cattle slaughtered should be increased. Most importantly, the grading data of carcass yields should be shared with the breeding farmers and be used for breeding optimization, which reduces the production costs.

Since the CBA in this study was performed based on assumptions, there are some limitations that require attention and deeper analyses. Assumptions made in the determination of persistent time, discount rate, system capabilities, breeding optimization, etc., could easily bias the analysis and deem it unrealistic. For this reason, comprehensive research on the variables needs to be undertaken and a thorough sensitivity analysis is required. While the research and sensitivity analysis performed for this study was acceptable, there is still room for improvement in further studies.

The yield results from the system can be used to build a useful database that is accessible to stakeholders and plays a vital role in decision making. This database can be used, for example, by governments to monitor carcass yield across the nation, to feed into the online auction system, predict beef carcass yield estimates prior to any further dissection, etc.

This study proves that the automation of the slaughter line is economically viable and can be employed for modernizing meat production in Korea. Since this is developed domestically, the initial costs of the system and the repair and maintenance costs are relatively low in relation to other renowned imaging-based carcass grading systems. As proved by an earlier economic analysis of an imported pork carcass grading system [27], it can be quite costly to procure and maintain such carcass grading systems, making them burdensome for some small-scale slaughterhouses. This renders the deployment of the system even more economically beneficial.

## Figures and Tables

**Table 1:** A summary of the fixed and variable costs incurred in the project.

Classification	Specifics	Computation	Total (KRW)
Fixed Costs	System cost	Price of procuring system	79,465,566
	System installation	Technical team (2 people) × daily wage (260,121) × 5 days = 2,601,210	2,601,210
	Overhead rail modifications	Cost of 10 m of overhead rail (7,030,000) + installation labor costs (1,253,000) = 8,283,000	8,283,000
	Cost of space/land occupied by system	Area required by system (17.5 m^2^) × Average price of land per m^2^ (196,657) = 3,441,498	3,441,498
Variable Costs	Operational costs	Electrical costs (15,160,339) + Internet costs (270,000) + administrative costs (12,624,009) = 28,054,348	28,054,348 per year
	Maintenance costs 1	RFID tags replacement (641,200) + Software updates (6,119,700) = 6,760,900	6,760,900 per year
	Maintenance costs 2	Repairs for PC and NAS (489,930 + 433,000) + Replacement cameras (6,299,593) = 7,222,523	7,222,523 in 4th and 8th years
Total fixed costs incurred at the beginning of the project			93,791,273
Total variable costs incurred every year			34,815,248
Total variable costs incurred in 4th and 8th years			7,222,523

**Table 2:** Summary of the parameters and respective values considered for sensitivity analysis.

Parameter	Base Value	Values for Sensitivity Analysis
Discount rate	4.5%	2.5–6.5%, intervals of 0.5%
Initial investment	93.8 million KRW	−20–20%, intervals of 10%
Number of Employees relieved	1 employee	0, 1, 2
% Costs saved due to breeding practice optimization	0.5%	0%, 0.25%, 0.5%, 0.75% 1.0%
Avg. carcass throughput	2385 carcasses/year	2100–3500, intervals of 350

**Table 3:** A summary of the CBA of the project.

Particulars		Details
Analysis target: Online image-analysis beef carcass yield estimation system		
Costs	Fixed costs	System price + installation: 82,066,776 KRW
		Overhead rail adjustment: 8,283,000 KRW
		Land/space cost: 3,441,498 KRW
	Variable costs	Operation costs: 28,054,348 KRW per year
		Maintenance costs: Varying amount
Benefits	Direct	Labor reduction: 67,631,460 KRW per year
	Indirect	Cost saved due to breeding optimization: Varying
	Others	Accessible online database
		Food safety improvement
		Worker safety
Analysis methods	NPV, IRR, BCR	
Discount rate	4.5%	
Sensitivity analysis	Change in discount rate (2.5–6.5%, intervals of 0.5%)	
	2. Initial investment (−20–20%, intervals of 10%)	
	3. Number of Employees let go (0, 1, 2)	
	4. Costs saved due to breeding optimizing (0.0%, 0.25%, 0.5%, 0.75%, 1%)	
	5. Avg. carcass throughput (2100 to 3500, intervals of 350)	

**Table 4:** Project cash flows (in KRW) and CBA results.

Period	Benefits	Costs	Depreciation Cost		Net Present Benefits		Net Present Cost	
Opening day	0	93,791,273	0		0		93,791,273	
Year 1	42,661,258	34,815,248	17,088,042		40,824,170		49,668,220	
Year 2	43,375,820	34,815,248	15,189,371		39,720,538		45,790,727	
Year 3	47,323,028	34,815,248	13,290,699		41,469,009		42,155,079	
Year 4	65,941,358	42,037,771	11,392,028		55,295,873		44,804,164	
Year 5	114,430,653	34,815,248	9,493,357		91,824,997		35,555,486	
Year 6	154,742,291	34,815,248	7,594,685		118,825,946		32,566,407	
Year 7	166,505,582	34,815,248	5,696,014		122,353,040		29,768,828	
Year 8	168,399,562	42,037,771	3,797,343		118,416,067		32,230,570	
Year 9	168,319,081	34,815,248	1,898,671		113,262,655		24,704,959	
Total	971,698,633	421,573,553	85,440,210		741,992,295		431,035,714	
**Results**	**NPV = 310,956,581**		**BCR = 1.72**			**IRR = 22.28%**		

## Data Availability

The data are available on request from the corresponding author.
